# Antibiotics in periodontal treatment: an umbrella review

**DOI:** 10.3389/fcimb.2025.1601464

**Published:** 2025-06-04

**Authors:** João Botelho, Patrícia Lyra, Gustavo G. Nascimento, Fábio R. M. Leite, José João Mendes, Vanessa Machado

**Affiliations:** ^1^ Egas Moniz Center for Interdisciplinary Research, Egas Moniz School of Health and Science, Almada, Portugal; ^2^ National Dental Research Institute Singapore, National Dental Centre Singapore, Queenstown, Singapore; ^3^ Oral Health Academic Clinical Programme, Duke-National University of Singapore (NUS) Medical School, Queenstown, Singapore

**Keywords:** periodontitis, antibiotics, periodontal treatment, systematic review, periodontal disease, antibiotic

## Abstract

**Aim:**

Antimicrobial stewardship envisions the appropriate use of antimicrobials, including antibiotics. Antibiotic therapy in Periodontology has been widely investigated over the years. This umbrella review aimed to appraise the methodological quality and meta-analytical strength and validity of the evidence of systematic reviews (SRs) on systemic and local antibiotics in periodontal therapy.

**Material and methods:**

After registration of the protocol (PROSPERO CRD42024527222), an extensive search, up to March 2024, for SRs that have assessed the effect of antibiotics in periodontal therapy, either nonsurgical and surgical, regardless of the types of patients and type of antibiotic. The methodological quality of SRs was judged using A MeaSurement Tool to Assess systematic Reviews 2. Fail-safe number of Rosenberg explored the number of nonsignificant, unpublished, or missing studies that would be required to change the direction of that evidence.

**Results:**

Forty-four SRs, consisting of 221 meta-analyses, were included. The overall methodological quality was low, with only four and two SRs of high or moderate quality, respectively. Out of 221 meta-analyses, 69 indicated that the effect of systemic or local antibiotics was statistically not significant. Twenty-nine meta-analyses from suggestive-to-strong strength from one high and three low methodological quality SRs indicated that the systemic or local antibiotics had a beneficial, statistically significant effect on periodontal health parameters, such as average clinical attachment loss, bleeding on probing or percentage of pocket closure. Of those, four strong evidence meta-analyses from a low-quality systematic review indicated significant and meta-analytically robust but with negligible effect. About 65.5% of the meta-analyses with suggestive to strong evidence are unlikely to change with more future studies.

**Conclusion:**

There is no robust evidence to support the use of antibiotics for periodontal management. Systemic antibiotics have a minimal effect on periodontitis and additional studies are unlikely to change the level of evidence.

## 1 Introduction

Periodontitis is a chronic inflammatory condition in which the dysbiosis of the subgingival microbiota plays a role (Hajishengallis and Chavakis, 2021). Effective therapies ought to result in considerable clinical improvements while limiting disease progression (Herrera et al., 2020; Teughels et al., 2020). After proper treatment, the oral microbiome is expected to shift towards eubiosis, marked by the reduction of dysbiotic pathogenic species and the re-establishment of a symbiotic microbial community compatible with health (Haffajee et al., 2006; Teles et al., 2006; Teles et al., 2013).

The ideal periodontal therapy should address the underlying causes of periodontitis, prioritizing the restoration of tissue homeostasis as the key factor in disease management while concurrently aiming to reduce the bacterial load. Establishing healthy conditions in the periodontal tissues creates a local environment that prevents the regrowth of periodontopathogenic bacteria and the recurrence of dysbiosis.

However, due to the complexities of modulating immune responses and the physiological processes of periodontal metabolism, most efforts have focused on reducing the bacterial load. This approach would, in theory, provide the periodontal tissues with an opportunity to return to equilibrium and initiate repair.

To overcome these challenges, adjunct therapies, particularly antibiotics, have been proposed as supplements to standard periodontal treatments. Hypothetically, antibiotics, administered locally or systemically, may help suppress pathogenic bacteria and promote clinical improvement (Keestra et al., 2015). Systemic antimicrobials have the advantage of reaching all oral surfaces and fluids, as well as potentially reaching tissues invaded by bacteria (Lavda et al., 2004; Lai et al., 2011). Local antibiotic therapy has lower adverse effects, greater patient adherence, and less risk of developing bacterial resistance (Herrera et al., 2020). A key challenge is determining whether, and in which cases, antibiotics will be beneficial, as there is still a lack of robust scientific evidence supporting their application. This is even more critical, considering the estimated 23.6% prevalence of severe periodontitis among dentate people worldwide (Trindade et al., 2023) underscoring the need for judicious use of antibiotics to avoid unnecessary risks in potentially billions of people. This goes in line with the current “antimicrobial stewardship” principles, which advocate for the careful and responsible management of antimicrobials to ensure their effective, responsible and appropriate use (World Health Organization, 2024).

Despite the literature suggesting potential benefits from adjunctive antimicrobial agents in periodontitis treatment, there is considerable heterogeneity in the types of antibiotics and protocols used. To address these issues, evidence-based studies can provide a comprehensive synthesis of existing research, aggregate data from multiple sources, and offer a high-level overview of the evidence. This approach can help identify consistent findings, highlight variations in study designs, and assess the overall quality and strength of evidence regarding the effectiveness of different antimicrobial agents. As such, we aimed to carry out an umbrella review to evaluate the methodological quality, meta-analytical strength and validity of the evidence presented in systematic reviews (SRs) on the use of systemic and local antibiotics in periodontal therapy.

## 2 Materials and methods

### 2.1 Protocol and reporting

The protocol was defined *a priori*, with details registered on PROSPERO (ID: CRD42024527222). The results are reported following the PRISMA guideline (**Appendix Table 1** in **Supplementary Material**) (Page et al., 2021).

### 2.2 Study selection

We searched on PubMed (via MEDLINE), Web of Science, EMBASE, CENTRAL (Cochrane) and LILACS studies published up to March 2024, without any language or publication date restrictions. We merged keywords and subject headings appropriately for each database using the following syntax: (antibiotic* OR antibact*) AND (periodont* OR gum OR Periodontal Diseases[MeSH]) AND (systematic review OR meta-analysis OR meta analysis of metaanalysis). In addition, grey literature was searched via http://www.opengrey.eu. Additional relevant literature was included after a manual search of the reference lists of the final included articles.

The electronic database search was carried out by two independent authors (J.B. and V.M.), and the final decision for inclusion was made according to the following criteria: (1) systematic reviews with meta-analysis; (2) results from human studies; (3) assessing the impact of the delivery of antibiotics (either systemic and/or local) as an adjunct of periodontal therapy.

As such, exclusion criteria were as follows: (1) systematic reviews without meta-analysis (that is, without pooled estimates) as these prevented the assessment of the meta-analytical quality; (2) systematic reviews reporting binary results without controls; (3) systematic reviews with meta-analysis that did not provide meta-analytic estimates and/or heterogeneity results; and (4) systematic reviews of systematic reviews (umbrella reviews). Additional *post-hoc* exclusion decisions of studies were made regarding some specificities found during the studies inclusion process: (a) systematic reviews restricted to studies from a particular country; and (b) secondary analysis of data sourced from previous systematic reviews.

### 2.3 Data extraction

We created a predefined table to obtain the necessary data from each eligible systematic review, including the study identification (authors and year), the number of studies included in the meta-analysis, type and number of studies included, antibiotic type, route of administration (systemic or local), population description, number of participants, number and type of meta-analysis (when applicable), methodological quality tool used, periodontal information collected, effect size and 95% CI, and funding information. Two independent researchers (J.B. and V.M.) extracted the information from each eligible systematic review, and all disagreements were resolved through a discussion with a third reviewer (P.L.). The agreement between the examiners was categorized as excellent (0.93, 95% CI: 0.89-0.97).

### 2.4 Methodological quality appraisal and grading of the evidence

Two expert reviewers, J.B. and V.M., evaluated the systematic reviews using the AMSTAR (A MeaSurement Tool to Assess systematic Reviews) 2 tool (Shea et al., 2017). The systematic reviews were classified into four categories based on their quality and following AMSTAR2 instructions: High (no or one minor weakness), Moderate (more than one minor weakness), Low (one major flaw with or without minor weaknesses), and Critically Low (more than one major flaw with or without minor weaknesses).

We graded meta-analyses following a previously published methodology (Papadimitriou et al., 2021). Significant associations were categorized into four evidence levels: strong, highly suggestive, suggestive, and weak evidence (Bellou et al., 2018; Papadimitriou et al., 2021). A category of strong evidence was attributed if all the following criteria were met: >1000 cases included in the meta-analysis, a threshold that provides 80% power for hazard ratios ≥1.20 (α=0.05) (Papadimitriou et al., 2021); a *P*-value ≤10^−6^ of statistical significance in valid meta-analysis (Sterne, 2001; Ioannidis et al., 2011; Johnson, 2013); heterogeneity (I^2^) below 50%; the 95% prediction interval excluded; and, no evidence of small study effects and excess significance bias. Highly suggestive evidence was set if: meta-analyses with >1000 cases; a random effects *P*-value ≤10^−6^, and the largest study in the meta-analysis was statistically significant. Suggestive evidence was defined if: meta-analyses with >1000 cases, random-effects *P*-value ≤ 10^−3^ were categorized (Sterne, 2001; Ioannidis et al., 2011; Johnson, 2013). If the latter conditions were not verified, the meta-analysis was classified as weak evidence.

Lastly, we combined AMSTAR-2 categories with meta-analytical strength into a single categorization, called “Overall Grading” as follows: Strong – if strong meta-analytical estimates and high methodological quality according to AMSTAR 2; Highly Suggestive – if highly suggestive meta-analytical estimates and high methodological quality according to AMSTAR 2 or if strong meta-analytical estimates and moderate methodological quality according to AMSTAR 2; Suggestive – if suggestive meta-analytical estimates and high methodological quality according to AMSTAR 2 or if highly suggestive meta-analytical estimates and moderate methodological quality according to AMSTAR 2; Weak - If the latter conditions were not verified, the meta-analysis was classified as weak evidence or if the SR is of low or very low quality.

### 2.5 Calculation of FSN

In nominally statistically significant meta-analyses, we determined the number of future studies of average null effect and average weight needed to detect a non-statistically significant summary estimate by calculating Rosenberg’s FSN (Rosenberg, 2005). We used the Meta-Essentials packages for binary (odds ratio, risk ratio, hazard ratio, incidence ratio or ratio of means) and continuous measures (mean difference, standardized mean difference or weighted mean difference) (Suurmond et al., 2017). We then calculated the median and range for each evidence grade (strong, highly suggestive, suggestive and weak).

### 2.6 Data handling and management

All data were collected in MS Office 365. Inferential statistical analyses were computed using R version 4.03.

## 3 Results

### 3.1 Selection and characteristics of the included meta-analyses

**Figure 1:**
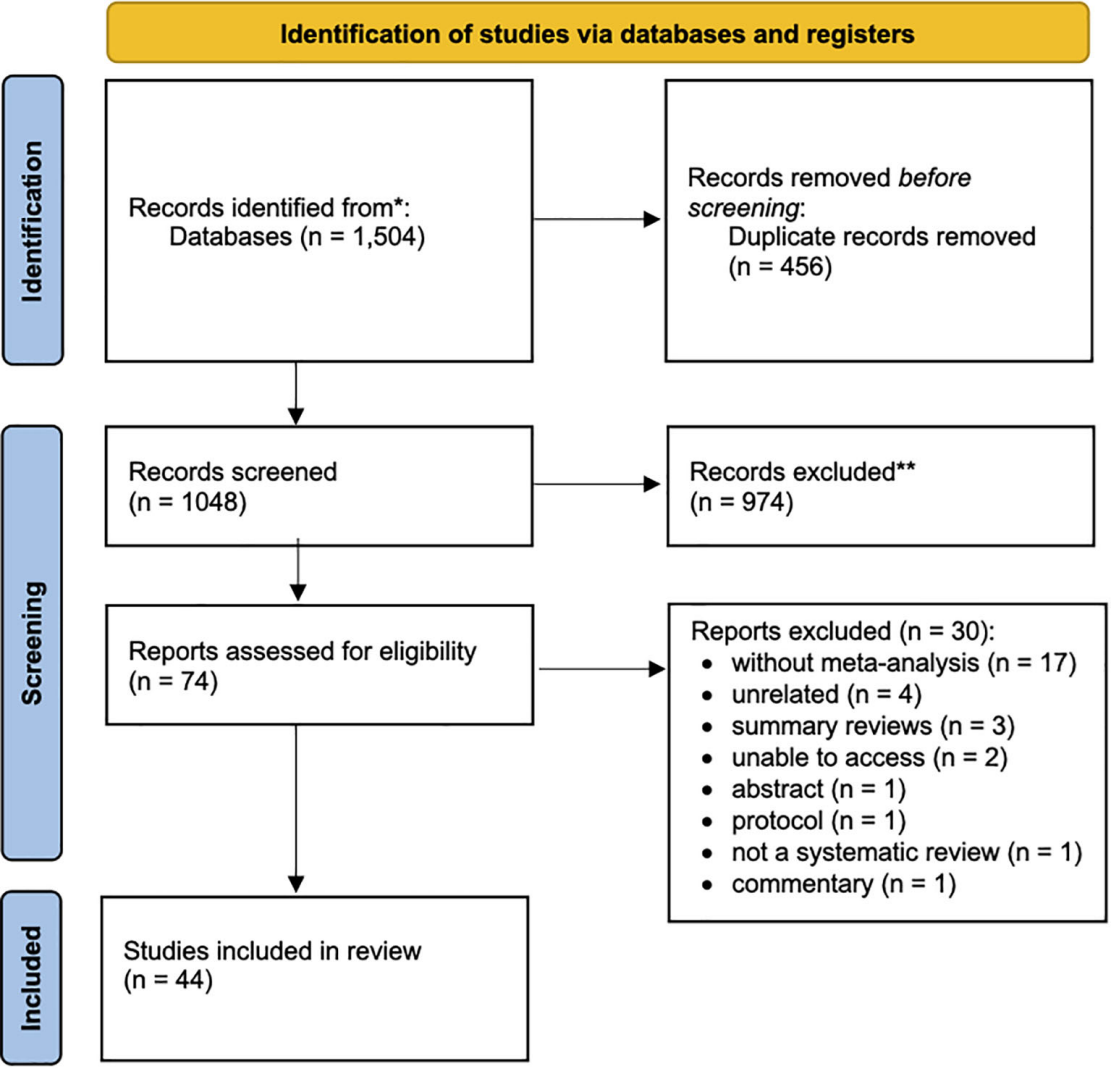
PRISMA Flowchart.

Our search retrieved a total of 1,544 entries (**Figure 1**). After removing duplicates (n=456), a total of 1,088 records were screened for titles and abstracts against the eligibility criteria. After judging the full-paper of 74 records, thirty studies were excluded (the list of excluded studies with justification for exclusion is detailed in **Appendix Table 2** in **Supplementary Material**). Excellent inter-examiner reliability was confirmed at the full-text screening (Cohen’s kappa score = 0.94, 95% CI: 0.92; 0.96). A final sample of 44 SRs were included for quantitative and qualitative appraisal (Elter et al., 1997; Herrera et al., 2002; Hung and Douglass, 2002; Pavia et al., 2003; Pavia et al., 2004; Bonito et al., 2005; Bono and Brunotto, 2010; Sgolastra et al., 2011; Moreno Villagrana and Gómez Clavel, 2012; Sgolastra et al., 2012a; Sgolastra et al., 2012b; Matesanz-Pérez et al., 2013; Zandbergen et al., 2013; Kolakovic et al., 2014; Sgolastra et al., 2014; Keestra et al., 2015; Rabelo et al., 2015; Santos et al., 2015; Chambrone et al., 2016; Grellmann et al., 2016; Nadig and Shah, 2016; Renatus, 2016; Rovai et al., 2016; Zhang et al., 2016; Assem et al., 2017; Lira Junior et al., 2017; McGowan et al., 2018; Souto et al., 2018; Nibali et al., 2019; Yap and Pulikkotil, 2019; Zheng et al., 2019; Herrera et al., 2020; Munasur et al., 2020; Nath et al., 2020; Teughels et al., 2020; Wang et al., 2020; Yusri et al., 2021; Zhao et al., 2021; Karrabi et al., 2022; Kherul Anuwar et al., 2022; Atieh et al., 2023; Tang et al., 2023; Wu et al., 2023; Zanatta et al., 2024).

Most studies reported results following the PRISMA statement (56.8%, n=25) or the QUality Of Reporting Of Meta-analyses (QUORUM) statement (n=2, 4.5%) (**Appendix Table 3** in **Supplementary Material**). Yet, 16 did not report following a reporting guideline for systematic reviews (36.4%). While for the risk of bias (ROB) (methodological quality), Cochrane ROB tools were the most used, either ROB (n=17, 38.6%) or ROB2 (n=8, 18.2%), or the Jadad Scale (n=2, 4.5%). Nevertheless, five SRs did not report the use of an appropriate instrument to assess methodological quality (11.4%).

Overall, 221 meta-analytic comparisons were included (**Appendix Table 4** in **Supplementary Material**). Except for six meta-analyses, nearly all (n=215; 97.3%) used a continuous effect size (**Supplementary Data 3**). Mean Difference (61.5%, n=136), Weighted Mean Difference (25.3%, n=56), and Standardized Mean Difference (7.7%, n=17) were the most commonly reported effect measures (**Appendix Table 4** in **Supplementary Material**). Most SRs focused only on systemic antibiotics (n=44, 72.1%), 11 on local antibiotics (n=11, 18.0%) and 6 on both systemic and local antibiotics (n=6, 9.8%).

### 3.2 Grading of meta-analytical evidence from the impact of antibiotics

Four meta-analyses from a single SR (Teughels et al., 2020) achieved strong evidence for the use of systemic antibiotics (combining multiple types) for average CAL (0.3 mm, 95% CI: 0.2;0.4), average CAL in moderately to deep pockets (0.4 mm, 95% CI: 0.3;0.5), average bleeding on probing (6.6%, 95% CI: 4.2;9.1) and average percentage of pocket closure (-14.5, 95% CI: -17.9;-11.1) (**Appendix Table 4** in **Supplementary Material**).

Fifteen estimates from three distinct SRs achieved highly suggestive evidence (Keestra et al., 2015; Herrera et al., 2020; Teughels et al., 2020). Two regarding the use of local antibiotics (combining multiple types) for mean probing pocket depth (PPD) and clinical attachment level (CAL) change. The remaining estimates concerned the use of systemic antibiotics (combining multiple types), for multiple clinical measures, such as average PPD (both overall, moderately deep pockets, deep pockets or change in frequency of multiple PPD thresholds).

Ten meta-analyses from a single SR (Keestra et al., 2015) obtained suggestive evidence for the use of systemic antibiotics (combining multiple antibiotics) for PPD, CAL and bleeding on probing (BoP) changes.

### 3.3 Methodological quality assessment

Inter-examiner reliability was very good (Cohen kappa score = 0.88; 95%CI: 0.84-0.92) for AMSTAR2. Four SRs were categorized as high (6.6%) and two as moderate (3.3%) methodological quality, according to the overall score rendered by AMSTAR 2 (**Appendix Table 4** in **Supplementary Material**). The majority presented critically low (n=40, 65.6%) to low methodological quality (n=15, 24.6%). The included SRs predominantly failed to report on the funding sources for the studies included (n=57, 93.4%), explain the selection of the study designs for inclusion in the review (n=50, 82.0%) and list the excluded studies with the respective justification (n=36, 59.0%). In addition, studies predominantly failed to account for the risk of bias in individual studies when interpreting or discussing the results (n=35, 57.4%). For studies conducting meta-analysis, there was often a lack of assessment of the potential impact of bias in individual studies on the results of the meta-analysis or other evidence synthesis (n=34, 55.7%). Although the median completeness of AMSTAR2 was 62.0%, the median completeness of critical domains was 46.8% (**Appendix Table 5** in **Supplementary Material**).

### 3.4 Number of additional studies needed to change current meta-analytic evidence

Three studies performed network meta-analyses (Wang et al., 2020; Kherul Anuwar et al., 2022; Zanatta et al., 2024) and eight did not report I^2^ scores (Elter et al., 1997; Herrera et al., 2002; Hung and Douglass, 2002; Pavia et al., 2003; Pavia et al., 2004; Moreno Villagrana and Gómez Clavel, 2012; Zandbergen et al., 2013; Renatus, 2016). Thus, those SRs were not included in this evaluation. Therefore, 215 meta-analyses from 33 SRs were assessed for their statistical strength (**Appendix Table 6** in **Supplementary Material**). Among the twenty-nine meta-analyses across three distinct studies, which cumulatively yielded suggestive to strong evidence, the median fail-safe number (FSN) amounted to 35 (with a range of 0 to 175). For each level of evidence, the median FSN was 57 (ranging from 35 to 160) for suggestive, 16 (ranging from 0 to 175) for highly suggestive, and 20 (ranging from 1 to 126) for strong evidence (as per **Appendix Table 5** in **Supplementary Material**). In 65.5% of the meta-analyses (n=19) for these evidence categories, the FSN was higher than the number of studies included, suggesting that the statistical significance of the summary estimates is unlikely to change as more studies are added in the future. Concerning the 186 meta-analyses with weak evidence, the median FSN was 0 (ranging from 0 to 50,567). In 16 comparisons (8.3%), the FSN was smaller than the number of studies included in the existing meta-analyses.

### 3.5 Overall grading

The combination of AMSTAR 2 with meta-analytical evidence grading showed that there is low-quality evidence. Despite the meta-analytical estimates having strong (n=4), highly suggestive (n=15) and suggestive (n=10) strength, the systematic reviews from which they originated had low methodological quality, thus nominally categorized as weak evidence. The remaining studies had weak meta-analytical strength (n=192).

## 4 Discussion

The present umbrella review assessed a total of 44 meta-analyses with a total sample of 221 comparisons. Systemic antibiotics, whose meta-analysis combined multiple types, have been found to be supported by weak evidence. The efficacy of local antibiotics cannot be confidently recommended for clinical application. However, if future systematic reviews employ a high methodological standard, the overall strength of the meta-analytical findings is not expected to change according to fail-safe number statistics.

In systematic reviews involving meta-analyses of antibiotics used in periodontal therapy, it was often observed that the number of participants for each specific type of antibiotic was relatively low. This limitation poses a challenge in drawing robust and definitive conclusions about the efficacy of individual antibiotics. As a result, researchers frequently combine data from various antibiotic types into a single meta-analysis to enhance the statistical power and robustness of the findings. In fact, the meta-analyses nominally categorized as suggestive to strong meta-analytical evidence were the ones combining multiple types of antibiotics. However, this approach can mask the nuanced effects and potential benefits or drawbacks of specific antibiotics. To address this issue and achieve a more accurate and detailed understanding of each antibiotic’s role in periodontal therapy, there is a pressing need to expand the number of studies, particularly longitudinal ones, focused on each specific antibiotic type. Increased research efforts in this direction will provide more reliable evidence and inform better clinical decision-making, ultimately improving patient outcomes.

Furthermore, while local antibiotic strategies offer a targeted approach to periodontal infection control, their effectiveness often appears to lag behind systemic regimens. Local antibiotics may provide high concentrations at the site of infection with fewer systemic side effects. Still, the limited number of high-quality studies makes it difficult to compare their efficacy to systemic antibiotics fully. This disparity underscores the need for more rigorous, comparative research to determine the optimal use of local versus systemic antibiotics in periodontal therapy. Only with a comprehensive body of evidence can clinicians make informed decisions that maximize patient benefits while minimizing risks.

The systematic reviews (SRs) included in this analysis demonstrate a relatively low level of methodological quality, as evidenced by the AMSTAR 2 ratings. Several key deficiencies were noted, and these findings highlight the necessity of improving methodological rigor in SRs to enhance the reliability and validity of their conclusions. Researchers should prioritize transparent reporting, but the editorial process should also accommodate a more rigorous and thorough process. Addressing these methodological weaknesses is crucial for advancing the quality of evidence synthesis in this field and ensuring that clinical recommendations are based on robust and reliable evidence.

In our observation, the overall clinical measures in these meta-analyses frequently relied on average values rather than “clinically applicable” metrics, such as the percentage of disease sites meeting certain threshold measures. Average clinical measures can obscure the concrete recovery of individual patients by smoothing over the variations and nuances present within the data. For example, an average improvement in pocket depth reduction or attachment level gain may suggest a moderate effect. Yet, it fails to reveal how many sites actually achieved clinically significant improvements or how many patients experienced meaningful health benefits. This approach can potentially mislead practitioners about the true efficacy of the interventions, as it does not highlight the distribution of responses across different disease sites or patient populations.

Using threshold measures, such as the percentage of disease sites achieving a predefined reduction in pocket depth or gain in attachment levels, could provide a clearer and more accurate picture of clinical outcomes. These objective values offer a more detailed understanding of how well an intervention performs across various scenarios and patient groups. By reporting the percentage of sites meeting clinically significant thresholds, researchers can convey the extent of meaningful recovery and the potential impact of treatments in a way that average values cannot. This method allows for a better assessment of treatment efficacy and identification of which interventions are most beneficial in achieving specific clinical goals, ultimately guiding more effective and targeted periodontal therapy practices.

### 4.1 Implications for practice and research

#### 4.1.1 For clinical practice

The shortcomings of current systematic reviews indicate the necessity for clinicians to rigorously assess the credibility of the evidence prior to applying it in clinical practice. Given that the great majority of the systematic reviews demonstrated low to critically low methodological quality, the likelihood of bias is heightened, thereby potentially affecting clinical decision-making. Clinicians should exercise caution when interpreting the average clinical measurements reported in meta-analyses, as these may obscure the true range and concrete results across diverse patient populations and disease clinical features. Instead, it is crucial to concentrate on more objective indicators, such as the percentage of disease sites attaining clinically meaningful improvements, in order to gain a clearer picture of treatment efficacy.

The comparison between local and systemic antibiotic strategies reveals a gap in the robustness of evidence favoring systemic regimens. However, this should not overshadow the potential benefits of local antibiotic applications, which require further investigation. Clinicians should consider the individual patient’s context, including the severity of periodontitis and potential side effects, when deciding between local and systemic antibiotic treatments.

#### 4.1.2 For future research

Based on these results, and with antibiotics having negligible impact on periodontitis clinical management, caution is required when developing future clinical studies. Since the antibiotics used are already broad-spectrum, we recommend an antibiotic stewardship as an effort to discourage antibiotics during periodontal therapy. Antibiotics should only be considered in aggressive forms of periodontitis and, wherever possible, studying *a priori* antibiotic efficacy. Thus, future studies focusing on such cases should adopt robust designs, including longitudinal randomized controlled trials with standardized outcome measures to allow comparability within studies. The biological mechanisms underpinning the use of local antibiotics shall be explored as well and define clear clinical scenarios where their application is warranted. This will help ensure therapeutic precision while minimizing antimicrobial resistance.

## 5 Conclusion

There is no robust evidence to support the use of antibiotics for periodontal management. In severe cases of periodontitis, antibiotic treatment may be warranted. However, it is advisable to evaluate the effectiveness of antibiotics beforehand when feasible.
