# Supplementary material for: Antibiotics in periodontal treatment: an umbrella review
Source: Front Cell Infect Microbiol. 2025 Jun 4;15:1601464. doi: 10.3389/fcimb.2025.1601464 (PMC12174147; doi:10.3389/fcimb.2025.1601464)
Supplement: Supplementary file 1 [file Table1.docx]

Appendix References

Assem NZ, Alves MLF, Lopes AB, Gualberto Junior EC, Garcia VG, Theodoro LH. 2017. Antibiotic therapy as an adjunct to scaling and root planing in smokers: a systematic review and meta-analysis. Braz Oral Res. 31(0). [accessed 2024 Jul 5]. http://www.scielo.br/scielo.php?script=sci_arttext&pid=S1806-83242017000100951&lng=en&tlng=en.

Atieh M, Shah M, Hakam A, Alghafri M, Tawse‐Smith A, Alsabeeha N. 2023 Oct 24. Systemic azithromycin versus amoxicillin/metronidazole as an adjunct in the treatment of periodontitis: a systematic review and meta‐analysis. Aust Dent J.:adj.12991.

Bonito AJ, Lux L, Lohr KN. 2005. Impact of Local Adjuncts to Scaling and Root Planing in Periodontal Disease Therapy: A Systematic Review. J Periodontol. 76(8):1227–1236.

Bono A, Brunotto M. 2010. Amoxicillin/metronidazole or scaling and root planing in the treatment of chronic periodontitis. Acta Odontol Latinoam AOL. 23(3):196–203.

Chambrone L, Vargas M, Arboleda S, Serna M, Guerrero M, De Sousa J, Lafaurie GI. 2016. Efficacy of Local and Systemic Antimicrobials in the Non‐Surgical Treatment of Smokers With Chronic Periodontitis: A Systematic Review. J Periodontol. 87(11):1320–1332.

Elter JR, Lawrence HP, Offenbacher S, Beck JD. 1997. Meta‐analysis of the effect of systemic metronidazole as an adjunct to scaling and root planing for adult periodontitis. J Periodontal Res. 32(6):487–496.

Grellmann AP, Sfreddo CS, Maier J, Lenzi TL, Zanatta FB. 2016. Systemic antimicrobials adjuvant to periodontal therapy in diabetic subjects: a meta‐analysis. J Clin Periodontol. 43(3):250–260.

Herrera D, Matesanz P, Martín C, Oud V, Feres M, Teughels W. 2020. Adjunctive effect of locally delivered antimicrobials in periodontitis therapy: A systematic review and meta‐analysis. J Clin Periodontol. 47(S22):239–256.

Herrera D, Sanz M, Jepsen S, Needleman I, Roldán S. 2002. A systematic review on the effect of systemic antimicrobials as an adjunct to scaling and root planing in periodontitis patients. J Clin Periodontol. 29(s3):136–159.

Hung H, Douglass CW. 2002. Meta‐analysis of the effect of scaling and root planing, surgical treatment and antibiotic therapies on periodontal probing depth and attachment loss. J Clin Periodontol. 29(11):975–986.

Karrabi M, Baghani Z, Venskutonis T. 2022. Amoxicillin/Metronidazole Dose Impact as an Adjunctive Therapy for stage II - III grade C Periodontitis (Aggressive Periodontitis) at 3- And 6-Month Follow-Ups: a Systematic Review and Meta-Analysis. J Oral Maxillofac Res. 13(1). [accessed 2024 Jul 5]. http://www.ejomr.org/JOMR/archives/2022/1/e2/v13n1e2ht.htm.

Keestra JAJ, Grosjean I, Coucke W, Quirynen M, Teughels W. 2015. Non‐surgical periodontal therapy with systemic antibiotics in patients with untreated chronic periodontitis: a systematic review and meta‐analysis. J Periodontal Res. 50(3):294–314.

Kherul Anuwar AH, Saub R, Safii SH, Ab-Murat N, Mohd Taib MS, Mamikutty R, Ng CW. 2022. Systemic Antibiotics as an Adjunct to Subgingival Debridement: A Network Meta-Analysis. Antibiotics. 11(12):1716.

Kolakovic M, Held U, Schmidlin PR, Sahrmann P. 2014. An estimate of pocket closure and avoided needs of surgery after scaling and root planing with systemic antibiotics: a systematic review. BMC Oral Health. 14(1):159.

Lira Junior R, Santos CDMM, Oliveira BH, Fischer RG, Santos APP. 2017. Effects on HbA1c in diabetic patients of adjunctive use of systemic antibiotics in nonsurgical periodontal treatment: A systematic review. J Dent. 66:1–7.

Matesanz‐Pérez P, García‐Gargallo M, Figuero E, Bascones‐Martínez A, Sanz M, Herrera D. 2013. A systematic review on the effects of local antimicrobials as adjuncts to subgingival debridement, compared with subgingival debridement alone, in the treatment of chronic periodontitis. J Clin Periodontol. 40(3):227–241.

McGowan K, McGowan T, Ivanovski S. 2018. Optimal dose and duration of amoxicillin‐plus‐metronidazole as an adjunct to non‐surgical periodontal therapy: A systematic review and meta‐analysis of randomized, placebo‐controlled trials. J Clin Periodontol. 45(1):56–67.

Moreno Villagrana AP, Gómez Clavel JF. 2012. Antimicrobial or Subantimicrobial Antibiotic Therapy as an Adjunct to the Nonsurgical Periodontal Treatment: A Meta-Analysis. ISRN Dent. 2012:1–11.

Munasur SL, Turawa EB, Chikte UME, Musekiwa A. 2020. Mechanical Debridement with Antibiotics in the Treatment of Chronic Periodontitis: Effect on Systemic Biomarkers―A Systematic Review. Int J Environ Res Public Health. 17(15):5601.

Nadig P, Shah M. 2016. Tetracycline as local drug delivery in treatment of chronic periodontitis: A systematic review and meta-analysis. J Indian Soc Periodontol. 20(6):576.

Nath S, Pulikkotil S, Dharmarajan L, Arunachalam M, Jing K. 2020. Effect of locally delivered doxycycline as an adjunct to scaling and root planing in the treatment of periodontitis in smokers: A systematic review of randomized controlled trials with meta-analysis and trial sequential analysis. Dent Res J. 17(4):235.

Nibali L, Koidou VP, Hamborg T, Donos N. 2019. Empirical or microbiologically guided systemic antimicrobials as adjuncts to non‐surgical periodontal therapy? A systematic review. J Clin Periodontol. 46(10):999–1012.

Pavia M, Nobile CGA, Angelillo IF. 2003. Meta‐Analysis of Local Tetracycline in Treating Chronic Periodontitis. J Periodontol. 74(6):916–932.

Pavia M, Nobile CGA, Bianco A, Angelillo IF. 2004. Meta‐Analysis of Local Metronidazole in the Treatment of Chronic Periodontitis. J Periodontol. 75(6):830–838.

Rabelo CC, Feres M, Gonçalves C, Figueiredo LC, Faveri M, Tu Y, Chambrone L. 2015. Systemic antibiotics in the treatment of aggressive periodontitis. A systematic review and a Bayesian Network meta‐analysis. J Clin Periodontol. 42(7):647–657.

Renatus A. 2016. Clinical Efficacy of Azithromycin as an Adjunctive Therapy to Non-Surgical Periodontal Treatment of Periodontitis: A Systematic Review and Meta-Analysis. J Clin Diagn Res. [accessed 2024 Jul 5]. http://jcdr.net/article_fulltext.asp?issn=0973-709x&year=2016&volume=10&issue=7&page=ZE01&issn=0973-709x&id=8115.

Rovai ES, Souto MLS, Ganhito JA, Holzhausen M, Chambrone L, Pannuti CM. 2016. Efficacy of Local Antimicrobials in the Non‐Surgical Treatment of Patients With Periodontitis and Diabetes: A Systematic Review. J Periodontol. 87(12):1406–1417.

Santos CMML, Lira-Junior R, Fischer RG, Santos APP, Oliveira BH. 2015. Systemic Antibiotics in Periodontal Treatment of Diabetic Patients: A Systematic Review. Murdoch C, editor. PLOS ONE. 10(12):e0145262.

Sgolastra F, Gatto R, Petrucci A, Monaco A. 2012. Effectiveness of Systemic Amoxicillin/Metronidazole as Adjunctive Therapy to Scaling and Root Planing in the Treatment of Chronic Periodontitis: A Systematic Review and Meta‐Analysis. J Periodontol. 83(10):1257–1269.

Sgolastra F, Petrucci A, Gatto R, Giannoni M, Monaco A. 2011. Long‐Term Efficacy of Subantimicrobial‐Dose Doxycycline as an Adjunctive Treatment to Scaling and Root Planing: A Systematic Review and Meta‐Analysis. J Periodontol. 82(11):1570–1581.

Sgolastra F, Petrucci A, Gatto R, Monaco A. 2012. Effectiveness of Systemic Amoxicillin/Metronidazole as an Adjunctive Therapy to Full‐Mouth Scaling and Root Planing in the Treatment of Aggressive Periodontitis: A Systematic Review and Meta‐Analysis. J Periodontol. 83(6):731–743.

Sgolastra F, Severino M, Petrucci A, Gatto R, Monaco A. 2014. Effectiveness of metronidazole as an adjunct to scaling and root planing in the treatment of chronic periodontitis: a systematic review and meta‐analysis. J Periodontal Res. 49(1):10–19.

Souto MLS, Rovai ES, Ganhito JA, Holzhausen M, Chambrone L, Pannuti CM. 2018. Efficacy of systemic antibiotics in nonsurgical periodontal therapy for diabetic subjects: a systematic review and meta-analysis. Int Dent J. 68(4):207–220.

Tang Z, Fan Q, Jiang Q, Li X, Wang Y, Long H, Lai W, Jian F. 2023. The effect of antibiotics on the periodontal treatment of diabetic patients with periodontitis: A systematic review and meta-analysis. Front Pharmacol. 14:1013958.

Teughels W, Feres M, Oud V, Martín C, Matesanz P, Herrera D. 2020. Adjunctive effect of systemic antimicrobials in periodontitis therapy: A systematic review and meta‐analysis. J Clin Periodontol. 47(S22):257–281.

Wang C, Yang Y, Li H, Lin P, Su Y, Kuo MY, Tu Y. 2020. Adjunctive local treatments for patients with residual pockets during supportive periodontal care: A systematic review and network meta‐analysis. J Clin Periodontol. 47(12):1496–1510.

Wu S-Y, Wu C-Y, Lin L-Y, Chen Y, Huang H-Y, Lai Y-L, Lee S-Y. 2023. Systemic antibiotics adjuvants to scaling and root planing in type 2 diabetic and periodontitis individuals: Systematic review with network meta-analysis. Jpn Dent Sci Rev. 59:167–178.

Yap KCH, Pulikkotil SJ. 2019. Systemic doxycycline as an adjunct to scaling and root planing in diabetic patients with periodontitis: a systematic review and meta-analysis. BMC Oral Health. 19(1):209.

Yusri S, Elfana A, Elbattawy W, Fawzy El-Sayed KM. 2021. Effect of locally delivered adjunctive antibiotics during surgical periodontal therapy: a systematic review and meta-analysis. Clin Oral Investig. 25(9):5127–5138.

Zanatta FB, Antoniazzi RP, Oliveira LM, Lietzan AD, Miguez PA, Susin C. 2024. The efficacy of combining adjuvants with non‐surgical periodontal therapy in individuals with type 2 diabetes: A Bayesian network meta‐analysis. J Clin Periodontol. 51(5):610–630.

Zandbergen D, Slot DE, Cobb CM, Van Der Weijden FA. 2013. The Clinical Effect of Scaling and Root Planing and the Concomitant Administration of Systemic Amoxicillin and Metronidazole: A Systematic Review. J Periodontol. 84(3):332–351.

Zhang Y-H, Sun Y, Ma T, Zhao J. 2016. Efficacy of Systemic Antibiotic Combined with Non-surgical Periodontal Therapy for Aggressive Periodontitis: A Meta-analysis. Chin J Evid-Based Med. 16(5):585–591.

Zhao H, Hu J, Zhao L. 2021. The effect of drug dose and duration of adjuvant Amoxicillin-plus-Metronidazole to full-mouth scaling and root planing in periodontitis: a systematic review and meta-analysis. Clin Oral Investig. 25(10):5671–5685.

Zheng T, Bai Y, Zheng J, Liu H, Guo Z, Wang P. 2019. Efficacies of clarithromycin and metronidazole in treatment of chronic periodontitis: A Meta-analysis. J Jilin Univ Ed. 45(01):130.
