# Supplementary material for: Antibiotics in periodontal treatment: an umbrella review
Source: Front Cell Infect Microbiol. 2025 Jun 4;15:1601464. doi: 10.3389/fcimb.2025.1601464 (PMC12174147; doi:10.3389/fcimb.2025.1601464)
Supplement: Supplementary file 3 [file Table3.docx]

Supplementary Data 2 – List of excluded studies with reasons

| 1 | Smiley CJ and Tracy SL and Abt E and Michalowicz BS and John MT and Gunsolley J and Cobb CM and Rossmann J and Harrel SK and Forrest JL and Hujoel PP and Noraian KW and Greenwell H and Frantsve-Hawley J and Estrich C and Hanson N Systematic review and meta-analysis on the nonsurgical treatment of chronic periodontitis by means of scaling and root planing with or without adjuncts. 2015 7 Journal of the American Dental Association (1939). doi:10.1016/j.adaj.2015.01.028 | Unrelated |
| --- | --- | --- |
| 2 | Souza EQM and da Rocha TE and Toro LF and Guiati IZ and Ervolino E and Garcia VG and Wainwright M and Theodoro LH Antimicrobial photodynamic therapy compared to systemic antibiotic therapy in non-surgical treatment of periodontitis: Systematic review and meta-analysis. 2020 9 Photodiagnosis and photodynamic therapy. doi:10.1016/j.pdpdt.2020.101808 | Unrelated |
| 3 | Liew AK and Punnanithinont N and Lee YC and Yang J Effect of non-surgical periodontal treatment on HbA1c: a meta-analysis of randomized controlled trials. 2013 9 Australian dental journal. doi:10.1111/adj.12091 | Unrelated |
| 4 | Goyal L and Gupta S and Samujh T Does nonsurgical periodontal therapy improve glycemic control? 2023 3 Evidence-based dentistry. doi:10.1038/s41432-023-00860-0 | Unrelated |
| 5 | Rajendra A and Spivakovsky S Antibiotics in aggressive periodontitis, is there a clinical benefit? 2016 12 Evidence-based dentistry. doi:10.1038/sj.ebd.6401197 | Summary review |
| 6 | Martín-Cabezas R and Huck O No evidence to support benefit of 14-day courses of amoxicillin-plus-metronidazole as adjunct to non-surgical periodontal treatment at three months. 2019 3 Evidence-based dentistry. doi:10.1038/s41432-019-0013-x | Summary review |
| 7 | Shelswell J The clinical benefit of adjunctive antibiotics alongside non-surgical periodontal therapy with respect to periodontopathogenic bacteria? 2019 12 Evidence-based dentistry. doi:10.1038/s41432-019-0061-2 | Summary review |
| 8 | Liu QY and Zhang Y and Li J and Xiang XR [Locally delivered macrolides as an adjunct to non-surgical periodontal treatment of chronic periodontitis: a meta analysis]. 2019 Shanghai kou qiang yi xue = Shanghai journal of stomatology. doi: | Unable to access |
| 9 | Albandar JM Adjunctive antibiotics with nonsurgical periodontal therapy improve the clinical outcome of chronic periodontitis in current smokers. 2012 9 The journal of evidence-based dental practice. doi:10.1016/S1532-3382(12)70015-5 | Commentary/Analysis |
| 10 | Paterno Holtzman, L. and Valente, N.A. and Vittorini Orgeas, G. and Copes, L. and Tomasi, C. and Clementini, M. and De Sanctis, M. Stability of clinical parameters after subgingival instrumentation, with or without different adjunctive interventions (step 2 of therapy), for the treatment of periodontitis 2022 J. Clin. Periodontol.. doi:10.1111/jcpe.13636 | Abstract |
| 11 | Sneha, V. and Bhuvaneshwarri, J. and Gita, B. Clinical efficacy of locally delivered minocycline in periodontitis – A systematic review 2020 Eur. J. Mol. Clin. Med.. doi: | Unable to access. Original site has an error |
| 12 | Khattri, S. and Arora, A. and Sumanth, K.N. and Prashanti, E. and Bhat, K.G. and Kusum, C.K. and Johnson, T.M. and Lodi, G. Adjunctive systemic antimicrobials for the non-surgical treatment of chronic and aggressive periodontitis 2017 Cochrane Database Syst. Rev.. doi:10.1002/14651858.CD012568 | Protocol |
| 13 | Pretzl B and Sälzer S and Ehmke B and Schlagenhauf U and Dannewitz B and Dommisch H and Eickholz P and Jockel-Schneider Y Administration of systemic antibiotics during non-surgical periodontal therapy-a consensus report. 2019 7 Clinical oral investigations. doi:10.1007/s00784-018-2727-0 | Not a systematic review |
| 14 | Khattri S and Kumbargere Nagraj S and Arora A and Eachempati P and Kusum CK and Bhat KG and Johnson TM and Lodi G Adjunctive systemic antimicrobials for the non-surgical treatment of periodontitis. 2020 11 The Cochrane database of systematic reviews. doi:10.1002/14651858.CD012568.pub2 | No meta-analysis |
| 15 | Hammami C and Nasri W Antibiotics in the Treatment of Periodontitis: A Systematic Review of the Literature. 2021 International journal of dentistry. doi:10.1155/2021/6846074 | No meta-analysis |
| 16 | O'Rourke VJ Azithromycin as an adjunct to non-surgical periodontal therapy: a systematic review. 2017 3 Australian dental journal. doi:10.1111/adj.12448 | No meta-analysis |
| 17 | Santos RS and Macedo RF and Souza EA and Soares RS and Feitosa DS and Sarmento CF The use of systemic antibiotics in the treatment of refractory periodontitis: A systematic review. 2016 7 Journal of the American Dental Association (1939). doi:10.1016/j.adaj.2016.02.013 | No meta-analysis |
| 18 | Angaji M and Gelskey S and Nogueira-Filho G and Brothwell D A systematic review of clinical efficacy of adjunctive antibiotics in the treatment of smokers with periodontitis. 2010 11 Journal of periodontology. doi:10.1902/jop.2010.100192 | No meta-analysis |
| 19 | Tan OL and Safii SH and Razali M Clinical Efficacy of Repeated Applications of Local Drug Delivery and Adjunctive Agents in Nonsurgical Periodontal Therapy: A Systematic Review. 2021 9 Antibiotics (Basel, Switzerland). doi:10.3390/antibiotics10101178 | No meta-analysis |
| 20 | Buset SL and Zitzmann NU and Weiger R and Walter C Non-surgical periodontal therapy supplemented with systemically administered azithromycin: a systematic review of RCTs. 2015 11 Clinical oral investigations. doi:10.1007/s00784-015-1499-z | No meta-analysis |
| 21 | Abdallaoui-Maan L and Bouziane A Effects of timing of adjunctive systemic antibiotics on the clinical outcome of periodontal therapy: A systematic review. 2020 3 Journal of clinical and experimental dentistry. doi:10.4317/jced.56324 | No meta-analysis |
| 22 | Mugri MH Efficacy of Systemic Amoxicillin-Metronidazole in Periodontitis Patients with Diabetes Mellitus: A Systematic Review of Randomized Clinical Trials. 2022 11 Medicina (Kaunas, Lithuania). doi:10.3390/medicina58111605 | No meta-analysis |
| 23 | Haffajee AD and Socransky SS and Gunsolley JC Systemic anti-infective periodontal therapy. A systematic review. 2003 12 Annals of periodontology. doi:10.1902/annals.2003.8.1.115 | No meta-analysis |
| 24 | Fritoli A and Gonçalves C and Faveri M and Figueiredo LC and Pérez-Chaparro PJ and Fermiano D and Feres M The effect of systemic antibiotics administered during the active phase of non-surgical periodontal therapy or after the healing phase: a systematic review. 2015 5 Journal of applied oral science : revista FOB. doi:10.1590/1678-775720140453 | No meta-analysis |
| 25 | Dilber E and Hagenfeld D and Ehmke B and Faggion CM Jr A systematic review on bacterial community changes after periodontal therapy with and without systemic antibiotics: An analysis with a wider lens. 2020 12 Journal of periodontal research. doi:10.1111/jre.12803 | No meta-analysis |
| 26 | Zandbergen D and Slot DE and Niederman R and Van der Weijden FA The concomitant administration of systemic amoxicillin and metronidazole compared to scaling and root planing alone in treating periodontitis: =a systematic review=. 2016 2 BMC oral health. doi:10.1186/s12903-015-0123-6 | No meta-analysis |
| 27 | Garcia Canas P and Khouly I and Sanz J and Loomer PM Effectiveness of systemic antimicrobial therapy in combination with scaling and root planing in the treatment of periodontitis: a systematic review. 2015 3 Journal of the American Dental Association (1939). doi:10.1016/j.adaj.2014.12.015 | No meta-analysis |
| 28 | Kaufmann ME and Lenherr P and Walter C and Wiedemeier DB and Attin T and Schmidlin PR Systemically administered amoxicillin/ metronidazole versus azithromycin as adjuncts to subgingival instrumentation during non-surgical periodontal therapy. A systematic review. 2020 7 Swiss dental journal. doi: | No meta-analysis |
| 29 | Paterno Holtzman, L. and Valente, N.A. and Vittorini Orgeas, G. and Copes, L. and Tomasi, C. and Clementini, M. and De Sanctis, M. Stability of clinical parameters after subgingival instrumentation, with or without different adjunctive interventions (step 2 of therapy), for the treatment of periodontitis 2022 J. Clin. Periodontol.. doi:10.1111/jcpe.13636 | No meta-analysis |
| 30 | Ardila, C.-M. and Bedoya-García, J.-A. Clinical and Microbiological Efficacy of Adjunctive Systemic Quinolones to Mechanical Therapy in Periodontitis: A Systematic Review of the Literature 2022 Int. J. Dent.. doi:10.1155/2022/4334269 | No meta-analysis |
