# Supplementary material for: Antibiotics in periodontal treatment: an umbrella review
Source: Front Cell Infect Microbiol. 2025 Jun 4;15:1601464. doi: 10.3389/fcimb.2025.1601464 (PMC12174147; doi:10.3389/fcimb.2025.1601464)
Supplement: Supplementary file 4 [file Table4.docx]

Supplementary Data 3 – List of included studies in the present umbrella review

| Study | Country | Continent | Search Period | ATB the main focus of the SR | Antibiotic Type | Antibiotic | Reporting Guideline | RoB Tool | N studies included |
| --- | --- | --- | --- | --- | --- | --- | --- | --- | --- |
| Zanatta et al. (2024) | Brazil | South America | up to February 2023 | No | Systemic and Local | All | NR | ROB2 | 72 |
| Tang et al. (2023) | China | Asia | NR | Yes | Systemic and Local | All | PRISMA | ROB1 | 13 |
| Atieh et al. (2023) | United Arab Emirates | Asia | up to 21 May 2023 | Yes | Systemic | AZIT vs. AMOXI+MET | PRISMA | ROB2 | 5 |
| Wu et al. (2023) | Taiwan | Asia | up to October 11, 2021 | Yes | Systemic | All | PRISMA | ROB2 | 17 |
| Kherul Anuwar et al. (2022) | Malaysia | Asia | from 2000 onwards | Yes | Systemic | All | PRISMA | ROB2 | 30 |
| Karrabi et al. (2022) | Iran (Islamic Republic of) | Asia | until December 31, 2021 | Yes | Systemic | AMOX+MET | PRISMA | CONSORT | 6 |
| Yusri et al. (2021) | Egypt | Africa | Up to April 15, 2021 | Yes | Local | All | PRISMA | ROB2 | 9 |
| Zhao et al. (2021) | China | Asia | until May 31st, 2020 | Yes | Systemic | AMOX+MET | PRISMA | ROB1 | 11 |
| Wang et al. (2020) | Taiwan | Asia | until May 31st, 2020 | Yes | Local | All | PRISMA, Cochrane Collaboration, and Check Review checklists. | RoB 2 | 22 |
| Munasur et al. (2020) | South Africa | Africa | between January 2000 and October 2019 | Yes | Systemic | All | PRISMA | ROB1 | 19 |
| Teughels et al. (2020) | Belgium | Europe | NR | Yes | Systemic | All | NR | ROB1 | 34 |
| Herrera et al. (2020) | Spain | Europe | NR | Yes | Local | All | NR | ROB1 | 59 |
| Nath et al. (2020) | Australia | Oceania | till November 2018 | Yes | Local | Doxycycline | PRISMA | ROB2 | 5 |
| Nibali et al. (2019) | UK | Europe | up to 24 July 2018 | Yes | Systemic | All | PRISMA | ROB1 | 5 |
| Zheng et al. (2019) | China | Asia | from inception to June 2017 | Yes | Systemic | Clarithromycin + MET | NR | ROB1 | 5 |
| Yap et al. (2019) | Malaysia | Asia | until April 2018 | Yes | Systemic | Doxycycline | PRISMA | ROB2 | 6 |
| McGowan et al. (2018) | Australia | Oceania | Up to April 23, 2017 | Yes | Systemic | AMOX+MET | PRISMA | ROB1 | 18 |
| Souto et al. (2018) | Brazil | South America | up to August 2016 | Yes | Systemic | All | PRISMA | ROB1 | 11 |
| Assem et al. (2017) | Brazil | South America | From July 1994 to August 2016 | Yes | Systemic | All | PRISMA | Jadad Scale | 4 |
| Lira Junior et al. (2017) | Brazil | South America | through September 2016 | Yes | Systemic | All | NR | ROB1 | 8 |
| Renatus et al. (2016) | Germany | Europe | Up to May 2015 | Yes | Syst+Local | Azithromycin | PRISMA | ROB1 | 6 |
| Nadig and Shah (2016) | India | Asia | Up to January 31, 2017 | Yes | Local | Tetracycline | NR | ROB1 | 10 |
| Grellmann et al. (2016) | Brazil | South America | through May 2015 | Yes | Systemic | All | PRISMA | ROB2 | 14 |
| Chambrone et al. (2016) | Colombia | South America | through July 2015 | Yes | Syst+Local | All | PRISMA | ROB1 | 7 |
| Zhang et al. (2016) | China | Asia | from inception to January 2016 | Yes | Systemic | All | NR | ROB1 | 10 |
| Rovai et al. (2016) | Brazil | South America | up to January 2016 | Yes | Local | All | PRISMA | ROB1 | 6 |
| Rabelo et al. (2015) | Brazil | South America | up to June 2014 | Yes | Systemic | All | PRISMA, Cochrane Collaboration and Check Review | ROB1 | 14 |
| Keestra et al. (2015) | Belgium | Europe | until May 16, 2013 | Yes | Systemic | All | PRISMA | Self-developed | 43 |
| Santos et al. (2015) | Brazil | South America | through May 2015 | Yes | Systemic | All | NR | ROB1 | 18 |
| Kolakovic et al. (2014) | Switzerland | Europe | up to June 2013 | Yes | Systemic | All | PRISMA | NR | 20 |
| Sgolastra (2014) | Italy | Europe | through December 16, 2012 | Yes | Systemic | MET | PRISMA | CONSORT | 6 |
| Zandbergen et al. (2013) | The Netherlands | Europe | in the period through April 1, 2012. | Yes | Systemic | AMOX+MET | PRISMA | Self-developed | 28 |
| Matesanz-Pérez et al. (2013) | Spain | Europe | until July 2011 | Yes | Local | All | NR | ROB1 | 56 |
| Moreno Villagrana et al. (2012) | Mexico | North America | between 2001 and 2011 | Yes | Systemic | All | NR | None | 9 |
| Sgolastra et al. (2012) CP | Italy | Europe | through September 11, 2011 | Yes | Systemic | AMOX+MET | PRISMA | CONSORT | 4 |
| Sgolastra et al. (2012) AgP | Italy | Europe | through September 11, 2011 | Yes | Systemic | AMOX+MET | QUORUM | CONSORT | 6 |
| Sgolastra et al. (2011) | Italy | Europe | to November 1, 2010 | Yes | Systemic | Subantimicrobial-dose doxycycline | QUORUM | Self-developed | 3 |
| Bono and Brunotto (2010) | Argentina | South America | between January 1989 and January 2009 | Yes | Systemic | AMOX+MET | NR | NR | 10 |
| Bonito et al. (2005) | USA | North America | 1966 through December 2002 | Yes | Local | All | NR | Self-developed | 16 |
| Pavia et al. (2004) | Italy | Europe | published by June 2002 | Yes | Local | MET | NR | Chalmers et al. & Antczak et al. | 12 |
| Pavia et al. (2003) | Italy | Europe | up to December 2001 | Yes | Local | Tetracycline | NR | Chalmers et al. & Antczak et al. | 25 |
| Hung and Douglass (2002) | USA | North America | NR | Yes | Systemic | All | NR | NR | 8 |
| Herrera et al. (2002) | Spain | Europe | The search was limited to April 2001 | Yes | Systemic | All | Cochrane | Self-developed | 25 |
| Elter et al. (1997) | USA | North America | 1980-1995 | Yes | Systemic | MET | NR | NR | 16 |

ChP – Chronic Periodontitis

AgP – Aggressive Periodontitis

MET – Metronidazole

AZT – Azithromycin

AMX – Amoxicillin

NR – Not Reported

SD – subgingival debridement

SupraD – supragingival debridement

ATB – Antibiotic

SRP – Scaling and Root Planing
